# Strong Neutral Spatial Effects Shape Tree Species Distributions across Life Stages at Multiple Scales

**DOI:** 10.1371/journal.pone.0038247

**Published:** 2012-05-29

**Authors:** Yue-Hua Hu, Guo-Yu Lan, Li-Qing Sha, Min Cao, Yong Tang, Yi-De Li, Da-Ping Xu

**Affiliations:** 1 Key Laboratory of Tropical Forest Ecology, Xishuangbanna Tropical Botanical Garden, Chinese Academy of Sciences, Mengla, Yunnan, China; 2 Research Institute of Tropical Forestry, Chinese Academy of Forestry, Guangzhou, Guangdong, China; 3 Danzhou Key Field Station of Observation and Research for Tropical Agricultural Resources and Environments, Ministry of Agriculture, Danzhou, Hainan, China; 4 Rubber Research Institute, the Chinese Academy of Tropical Agricultural Sciences, Danzhou, Hainan, China; DOE Pacific Northwest National Laboratory, United States of America

## Abstract

Traditionally, ecologists use lattice (regional summary) count data to simulate tree species distributions to explore species coexistence. However, no previous study has explicitly compared the difference between using lattice count and basal area data and analyzed species distributions at both individual species and community levels while simultaneously considering the combined scenarios of life stage and scale. In this study, we hypothesized that basal area data are more closely related to environmental variables than are count data because of strong environmental filtering effects. We also address the contribution of niche and the neutral (i.e., solely dependent on distance) factors to species distributions. Specifically, we separately modeled count data and basal area data while considering life stage and scale effects at the two levels with simultaneous autoregressive models and variation partitioning. A principal coordinates of neighbor matrix (PCNM) was used to model neutral spatial effects at the community level. The explained variations of species distribution data did not differ significantly between the two types of data at either the individual species level or the community level, indicating that the two types of data can be used nearly identically to model species distributions. Neutral spatial effects represented by spatial autoregressive parameters and the PCNM eigenfunctions drove species distributions on multiple scales, different life stages and individual species and community levels in this plot. We concluded that strong neutral spatial effects are the principal mechanisms underlying the species distributions and thus shape biodiversity spatial patterns.

## Introduction

How large numbers of species coexist at a local scale (<1 km^2^) is a challenging question for ecologists. With the rapid improvement of computer technology and statistical tools, it is now feasible to integrate both niche and neutral processes into models to analyze species distribution data. Analytical methods, such as regression [1], ordination and machine learning, can be used to investigate the mechanisms underlying species coexistence [2], [3]. Traditionally, ecologists have used individual lattice count data to simulate species distributions at the individual species or community levels [4], [5]. In this method, trees are always counted as individuals regardless of factors such as age, size, branching and whether re-sprouting has occurred. However, the habitat associations of tree species may vary across life stages [6], [7], and thus, tree intensity variation across lattices may be insufficient to reflect species distribution patterns.

Many other traits of tree species can be used to simulate their distributions, such as percent cover, point quadrat frequency, biomass, basal area and energy and resource use [8]. These features may provide novel insights for understanding species distributions and their organizing mechanisms. We are unaware of previous work explicitly comparing the results of using these features and the results of using individual count data to model species distributions. Basal area, which represents tree size, plays a key role in determining the functional differences among species [9]. Basal area also correlates with biomass accumulation and reflects the ability of trees to compete for soil nutrients [10]. A comparative study in which individual count data and basal area data are examined separately will reveal the extent to which different results are generated by the two types of data.

Most species tend to be clumped in their dispersion pattern [11], which may cause strong spatial autocorrelation, i.e., greater or less similarity in variables located close to each other than would be expected if species were distributed randomly across geographic space [12]. This is commonly observed in species spatial distribution data [1]. To control Type I error rates and obtain good parameter estimates, it is necessary to use spatially explicit models in spatial analyses of species distributions [1], [12], [13]. In addition, environmental factors, such as topography and soil, are also widely considered in models of species distributions [14], [15]. Integrating spatial effects and environmental variables in species distribution models is generally accepted by ecologists [16], [17], [18].

The effects of life stage and scale are critical for analyzing spatial distributions of tree species. Physiological requirements, selective pressures and distribution patterns can vary across the life stages of plant species, which can lead to a shift in habitat preference throughout its ontogeny [19], [20], [21]. In fact, numerous empirical studies have identified that the mechanisms underlying tree species distributions do vary across different life stages in some forest dynamics plots [6], [7], [22], [23]. Similarly, previous studies point out that analyses results can differ at different scales in ecological studies [24], [25], indicating that the scale effect is important for tree species distributions [4], [5].

In this study, we modeled lattice count data and basal area data at the individual species level and at the community level while simultaneously considering scale and life stage effects. At the individual species level, a simultaneous autoregressive (SAR) model was used. The spatially autocorrelated variation in the error term of the SAR model is determined by cell connectivity, and the cell connectivity of the lattice basal area data and the count data is exactly the same based on cell positions. Therefore, the spatial structure should be identical for the basal area data and the count data. Under this premise, we hypothesized that basal area data are more closely related to environmental variables and predicted that the R-squared value of the fitted model based on basal area data would be higher than that based on count data because of strong environmental filtering effects. At the community level, we partitioned the variation in community composition between environmental variables and spatial effects for each of the two types of data. At this level, we also predicted that the variation explained by environmental variables would be higher for basal area data than for individual count data, also owing to strong environmental filtering effects.

## Materials and Methods

### Study Site and Data Collection

We analyzed tree species distributions within a 20-ha tropical forest dynamics plot (21°37′08″N, 101°35′ 07″E) in Xishuangbanna, Southwest China [26]. The community was an old-growth natural tropical seasonal rainforest tree community (more than 200 years old), but a small portion of the plot, located on the mountain ridge, was disturbed by humans approximately 40 years ago. The tree community was dominated by *Parashorea chinensis*, an emergent canopy species with a maximum height of approximately 60 m. Detailed descriptions of the climate, geology and flora of Xishuangbanna can be found in Cao et al. [27] and Zhu et al. [28]. The 20-ha plot was established in 2007, and a topographic survey was conducted of each node of a 10-m grid throughout the plot. All stems with a DBH (diameter at breast height) ≥1 cm were tagged, mapped, measured and identified. There were 468 tree and shrub species with individuals of DBH ≥1 cm in this plot [26].

To examine the mechanisms underlying any differences between the results obtained from the basal area and count data across life stages, we defined trees with DBH ≥1 cm as class 0. This class was itself divided into four DBH classes, representing different life stages of trees. This categorization of DBH classes followed He et al. [21]:
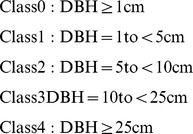



For a tree with multiple stems, we computed a proxy DBH and then classified the tree based on this proxy DBH. The calculation of proxy DBH followed Hu et al. [23].

To evaluate the influence of scale on species distribution, we grouped the trees within each DBH class using cells of 10×10 m, 20×20 m, 25×25 m and 50×50 m in size. This generated 20 combinations of DBH classes and cell sizes. Each DBH-cell size combination contained a group of tree species, and for each DBH-cell size combination, the tree species that occurred in at least 30 cells were chosen for regression analysis and variation partitioning (Table 1).

**Table 1:** Number of tree species in each of the 20 combinations of DBH and cell size.

DBH class	10×10 m	20×20 m	25×25 m	50×50 m
Class 0	206	192	187	153
Class 1	163	147	147	111
Class 2	70	61	56	33
Class 3	62	58	54	30
Class 4	25	22	21	10

Note: class 0 (DBH ≥1 cm), class 1 (1 cm ≤ DBH <5 cm), class 2 (5 cm ≤ DBH <10 cm), class 3 (10 cm≤ DBH <25 cm), class 4 (DBH ≥25 cm).

At each scale, the four topographic attributes of altitude, convexity, slope and aspect were calculated for each cell. These calculations followed Legendre et al. [5]. Third-degree polynomial equations were constructed with altitude, convexity and slope. The variables sin(aspect) and cos(aspect) were calculated from the aspect and used as explanatory variables. Finally, we obtained 11 expanded topographic variables. For 25×25 m cells, the altitudinal values at all nodes were interpolated by kriging the raw data from the 10×10 m cells.

Because soil attributes are crucial to species distributions, we collected 756 soil samples from throughout the 20-ha plot [23]. Nine soil attributes were analyzed, including available nitrogen, exchangeable potassium, extractable phosphorus, organic matter, soil pH, total potassium, total nitrogen, total phosphorus and soil bulk density, following the methods of Liu et al. [29]. For the soil attributes at each scale (cell size), the values at the four corners of each cell were interpolated by kriging from the 756 samples. After interpolation, the mean value of each soil attribute at the four corners of each cell was assigned as the value for that cell. This procedure was applied to each of the four scales of cell size. At each scale, we calculated the principal components from the mean values of the nine soil attributes and used only the first three components. Together, these first three components explained 84.5%, 83.5%, 86.9% and 89.1% of the total variation in soil attributes for the four cell sizes from 10×10 m to 50×50 m, respectively.

### The Simultaneous Autoregressive (SAR) Model

Guisan et al. [30] suggested that regression and ordination methods are both suited for species-specific and multiple species models. We chose the SAR model for the regression analyses of individual species because SAR has commonly been used for lattice summary data [31]. Specifically, the SAR spatial error model was used in this study in the following form:
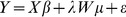
(1)


where *Y* is the response variable, in this case, the lattice count or basal area vector of a focal tree species at a particular cell size in a particular DBH class; *X* is the explanatory variable matrix constituted by the first three principal components of the soil variables and the 11 topographic variables at a particular cell size; *β* is a slope vector associated with the explanatory variables; *λ* is the spatial autoregressive coefficient; *μ* is a spatially dependent error term; ε is a random error term; and *W* is the spatial weighting matrix that indicates whether the cells are neighbors or not. The weight is defined as 1 if cells are immediate vertical and horizontal neighbors and 0 otherwise. For each focal cell, the cells sharing a common edge (border) with it were defined as its neighbor cells and were weighted by 1, and all other cells were weighted by 0. To avoid zero-inflated effects on the regression analysis, cells containing no trees were removed for each species. We found that the R-squared values obtained by fitting only the non-zero data were significantly higher than those obtained when the zero data were included.

To evaluate the relative importance of all of the explanatory variables in determining species distributions for each of the 20 combinations of DBH class and cell size for each type of data (lattice basal area and count), a principal component analysis (PCA) was used to analyze a transformed p-value matrix. The SAR model yields a p-value for each of the explanatory variables and *λ*, and the p-values for all species can be formatted as a matrix. Two such matrices were generated for each of the 20 DBH-cell size combinations: one that used basal area data and one that used count data. Because the p-values reflected the associations between responsible and explanatory variables in an inverse manner, the p-values themselves could not be used directly for the PCA. As a result, we performed a transformation procedure on the p-values to obtain the transformed p-value matrix, which positively reflected the association between responsible and explanatory variables and were suitable for PCA. The method used to transform the p-values followed Hu et al. [23]. Some of the p-values were small enough that a value of 0 was returned by the SAR model in the R statistical language [32], and the transformation procedure could not be applied to these p-values. To address this issue, p-values smaller than 10^−16^ were assigned a proxy value of 10^−16^. In each analysis, we plotted the scores of all of the explanatory variables on the first two principal component axes as arrows and assessed the relative importance of the explanatory variables based on their vector lengths.

### Community Composition Variation Partitioning

To quantify the contributions of the spatial and environmental variables to the variation in community composition observed for each of the two types of data, variation partitioning based on canonical redundancy analysis was applied [33]. The topographic and soil variables were grouped together as environmental variables for this analysis. To represent spatial variables, the principal coordinate neighbor matrix (PCNM) eigenfunctions were computed across all cells at each scale of cell size [5]. PCNMs with positive eigenvalues were retained, and forward selection (using a permutation test with 999 permutations and a 5% significance level) was used to identify the significant PCNMs. These selected PCNMs represented the spatial effects. We then partitioned the contributions of the environmental variables and the PCNMs. This procedure was repeated for each of the 20 DBH-cell size combinations for basal area data and count data.

To compare the R-squared values of the fitted regressive models as well as the total explained variation in community composition based on count data and basal area data, a Kruskal-Wallis rank-sum test was performed. We conducted SAR analyses and variation partitioning with the R (version 2.13.0) statistical language with the “errorsarlm” function of the “spdep” package and the “varpart” function of the “vegan” package, respectively [32].

## Results

**Figure 1:**
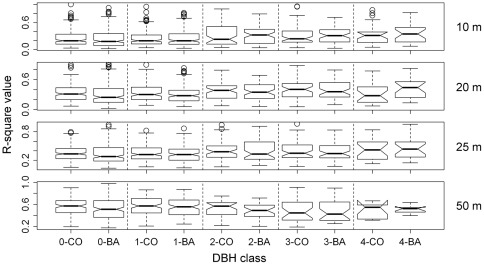
Distributions of the R-squared values of the fitted SAR models based on count data and basal area data for each of the 20 combinations of DBH and cell size. Each row represents a distinct scale of cell size; 0 to 4 in the x-axis labels represent DBH class 0 to 4, sequentially; “-CO” and “-BA” in the x-axis labels represent count data and basal area data, respectively. Classes 0 to 4 are defined as follows: class 0 (DBH ≥1 cm), class 1 (1 cm ≤ DBH <5 cm), class 2 (5 cm≤ DBH <10 cm), class 3 (10 cm ≤ DBH <25 cm) and class 4 (DBH ≥25 cm).

We found no significant differences in the R-squared value between the fitted SAR models based on the two types of data for any of the 20 DBH-cell size combinations, except for class 0 at the scales (cell sizes) of 20×20 m and 50×50 m (p-values of Kruskal-Wallis rank-sum test: 0.0087 and 0.0261, respectively). Among the 20 DBH-cell size combinations, the median R-squared value of the fitted models based on count data were greater than that based on basal area data in 14 cases, but only two of these cases were statistically significant. By contrast, basal area data generated greater R-squared values than count data in only 6 cases, and none of these differences were statistically significant. Fig. 1 illustrates the distributions of the R-squared values generated by the fitted SAR models based on each of the two types of data for the 20 DBH-cell size combinations.

**Figure 2:**
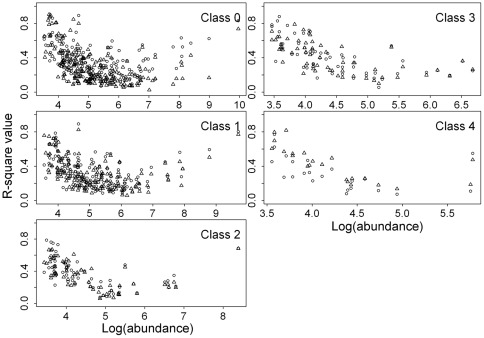
Relationships between the R-squared values of the fitted SAR models and total species abundance for each of the 5 DBH classes at the 20-m scale. Circles and triangles represent count data and basal area data, respectively. Classes 0 to 4 are defined as in Figure 1.

There was a positive trend in the R-squared value with increasing cell size (Fig. S1). However, there was no clear relationship between the R-squared value and the DBH class (Fig. S2). There was a negative relationship between the R-squared value and the total abundance of the studied species, except at the 50-m scale (Fig. 2, Figs. S3, S4, S5).

**Figure 3:**
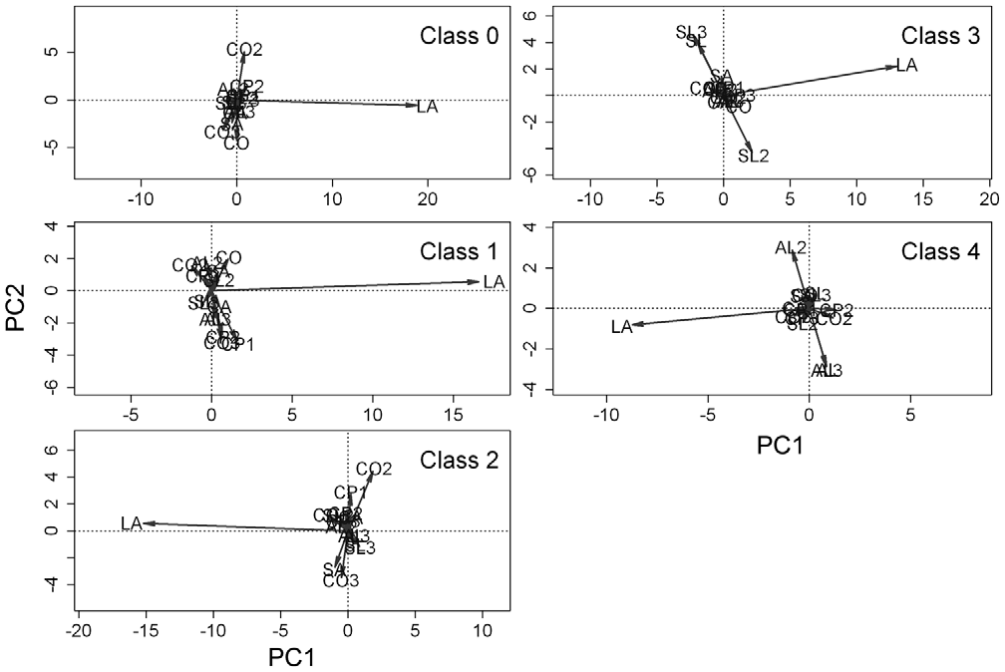
Principal component analysis ordinations (based on matrices of transformed p-values from the SAR models) of the 14 explanatory variables and the spatial autoregressive parameter *λ* for each of the 5 DBH classes at the 20-m scale of the count data. Classes 0 to 4 are defined as in Figure 1. The abbreviations in the third-degree polynomial equations of altitude, convexity and slope are as follows: altitude (AL), altitude^2^ (AL2), altitude^3^ (AL3), convexity (CO), convexity^2^ (CO2), convexity^3^ (CO3), slope (SL), slope^2^ (SL2) and slope3 (SL3). The abbreviations of the sine-cosine function of aspect and the spatial autoregressive parameter λ are as follows: cos(aspect) (CA), sin(aspect) (SA) and λ (LA). The abbreviations of the first three principal components of the soil variables are as follows: the first principal component (CP1), the second principal component (CP2) and the third principal component (CP3).

**Figure 4:**
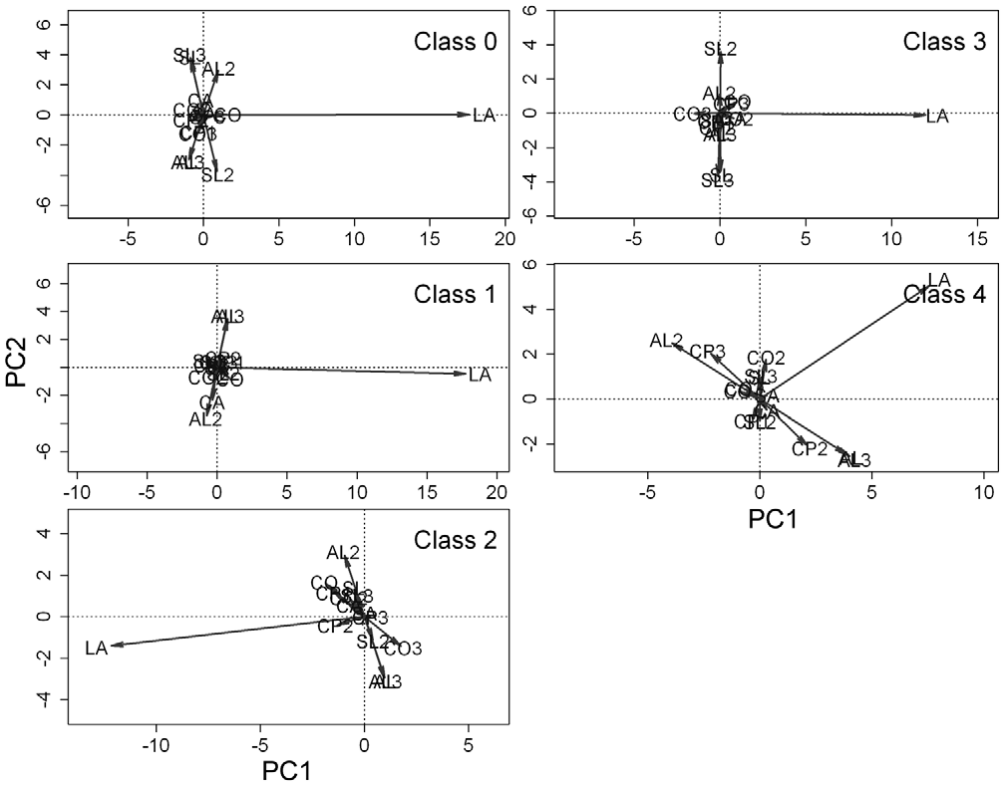
Principal component analysis ordinations (based on matrices of transformed p-values from the SAR models) of the 14 explanatory variables and the spatial autoregressive parameter λ for each of the 5 DBH classes at the 20-m scale, obtained with basal area data. Classes 0 to 4 are defined as in Figure 1. The abbreviations are defined as in Figure 3.

The spatial autoregressive parameter *λ* of the SAR fitted model had the longest vectors in the 20 DBH-cell size combinations for both count data and basal area data (Figs. 3 and 4, Figs. S6, S7, S8, S9, S10, S11), indicating that spatial effects played a more important role than any of the environmental variables in determining tree species distributions in this forest plot.

**Figure 5:**
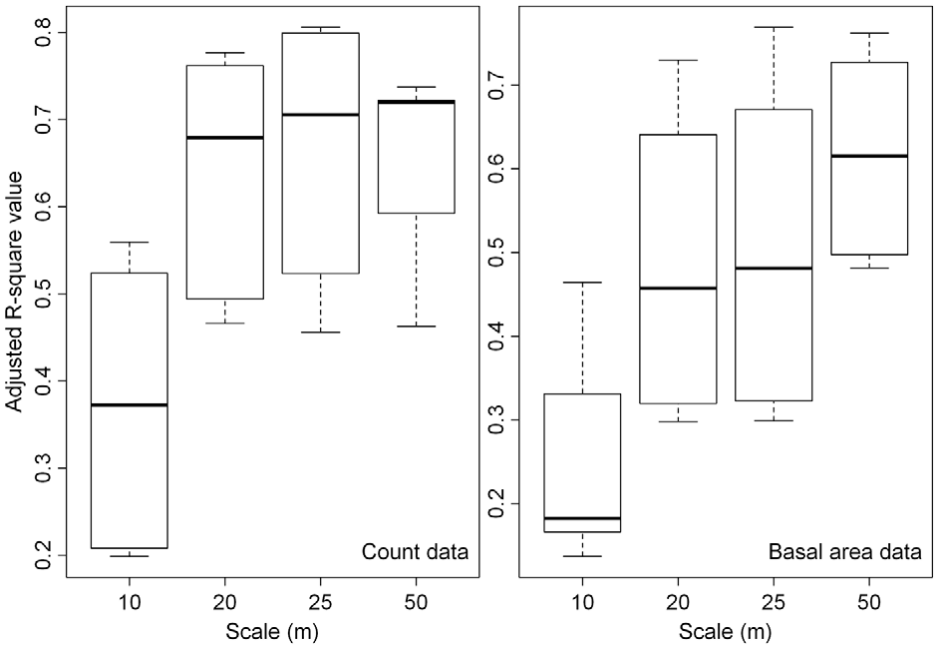
Distribution patterns of the total explained variation in community composition for each of the four scales of cell size based on count data and basal area data. The reduplicate data at each scale consisting of the 5 total explained variations of the 5 DBH classes of the variation partitioning results at each of the four scales.

The results of the community composition variation partitioning were consistent with the results generated by the regression analysis performed at the individual species level. Both analyses indicated that spatial effects are dominant in determining species distributions (Tables 2 and 3). There was no significant difference in the fraction of variation explained by the pure environmental variables when either the count data or the basal area data were used. For 10 of the 20 DBH-cell size combinations, the variation explained by the environmental variables was higher when basal area data were used than when count data were used. In addition, the Kruskal-Wallis rank-sum test revealed no significant difference in the total variation explained by the combined effects of spatial and environmental variables when either the basal area data or the count data were used. However, the count data yielded higher total explained variation than did the basal area data for 17 of the 20 DBH-cell size combinations. For both types of data, the total explained variation tended to increase as the scale (cell size) increased (Fig. 5). By contrast, for both types of data, the total explained variation decreased with an increase in the DBH size class (Fig. S12).

**Table 2:** Results of the partitioning variation between environmental variables and spatial effects for each of the 20 combinations of DBH and cell size using basal area data.

Cell size (m)	DBH class	[a] (%)	[b] (%)	[c] (%)	[d] (%)
10×10	Class 0	0.16	1.720	11.80	86.31
	Class 1	0.44	15.30	30.71	53.55
	Class 2	0.41	10.80	21.90	66.89
	Class 3	0.25	4.41	13.58	81.76
	Class 4	0.16	1.57	14.89	83.38
20×20	Class 0	0.98	9.45	19.37	70.20
	Class 1	0.49	31.57	40.92	27.02
	Class 2	0.62	27.90	35.55	35.94
	Class 3	0.60	16.08	29.03	54.29
	Class 4	0.71	9.18	22.08	68.04
25×25	Class 0	0.58	10.07	21.60	67.75
	Class 1	0.51	25.47	50.94	23.08
	Class 2	1.02	20.97	45.09	32.92
	Class 3	0.38	15.28	32.47	51.88
	Class 4	0.63	9.83	19.42	70.12
50×50	Class 0	6.85	19.94	22.94	50.27
	Class 1	1.79	33.66	40.75	23.80
	Class 2	2.21	37.14	33.35	27.30
	Class 3	10.46	21.74	15.97	51.83
	Class 4	8.35	15.82	37.35	38.48

Note: Adjusted R-squared statistics are shown. Fractions [a] – [d] are as follows: [a]  =  variation explained by the environmental variables and not spatially structured; [b]  =  variation explained by the environmental variables and spatially structured; [c]  =  spatially structured variation not explained by the environmental variables; [d]  =  residual variation. Fraction [b] is the intersection of the variation explained by linear models of the two groups of explanatory factors. Topographic and edaphic variables were used to compute fractions [a] and [b]. Principal coordinates of neighbor matrix eigenfunctions were used as explanatory variables to compute fractions [b] and [c]. class 0 (DBH ≥1 cm), class 1 (1 cm ≤ DBH <5 cm), class 2 (5 cm ≤ DBH <10 cm), class 3 (10 cm ≤ DBH <25 cm), class 4 (DBH ≥25 cm).

**Table 3:** Results of partitioning variation between environmental variables and spatial effects for each of the 20 combinations of DBH and cell size using count data.

Cell size (m)	DBH class	[a] (%)	[b] (%)	[c] (%)	[d] (%)
10×10	Class 0	0.58	15.83	39.51	44.08
	Class 1	0.47	13.28	38.65	47.60
	Class 2	0.54	12.86	23.86	62.74
	Class 3	0.28	4.95	15.60	79.17
	Class 4	0.22	3.99	15.68	80.12
20×20	Class 0	0.45	31.95	45.30	22.31
	Class 1	0.29	28.42	47.47	23.82
	Class 2	0.45	30.91	36.56	32.08
	Class 3	0.69	16.93	31.79	50.58
	Class 4	0.53	17.82	28.26	53.39
25×25	Class 0	0.49	23.96	56.15	19.40
	Class 1	−0.04	22.66	57.29	20.08
	Class 2	0.77	23.26	46.54	29.44
	Class 3	0.39	16.46	35.49	47.66
	Class 4	1.28	13.52	30.81	54.39
50×50	Class 0	2.66	31.52	39.54	26.27
	Class 1	2.26	28.89	40.78	28.07
	Class 2	5.43	35.39	31.38	27.80
	Class 3	3.41	29.35	26.47	40.76
	Class 4	6.60	20.79	18.88	53.73

Note: Adjusted R-squared statistics are shown. Fractions [a] – [d] are as follows: [a]  =  variation explained by the environmental variables and not spatially structured; [b]  =  variation explained by the environmental variables and spatially structured; [c]  =  spatially structured variation not explained by the environmental variables; [d]  =  residual variation. Fraction [b] is the intersection of the variation explained by linear models of the two groups of explanatory factors. Topographic and edaphic variables were used to compute fractions [a] and [b]. Principal coordinates of neighbor matrix eigenfunctions were used as explanatory variables to compute fractions [b] and [c]. class 0 (DBH ≥1 cm), class 1 (1 cm ≤ DBH <5 cm), class 2 (5 cm ≤ DBH <10 cm), class 3 (10 cm ≤ DBH <25 cm), class 4 (DBH ≥25 cm).

## Discussion

### The Contributions of Environmental Variables

Although environmental variables constrained a portion of the variations in the species distribution data, the variation partitioning results demonstrate that the environmental variables are strongly structured by PCNM eigenfunctions. In other words, the pure environmental variables play a limited role in determining species distributions, and most of the variations in environmental variables are derived from distance limitation. Among the environmental variables, the nonlinear topographic variables and the first two principal components of soil variables contribute more to species distributions than other environmental variables (Figs. 3 and 4, Figs. S6, S7, S8, S9, S10, S11). This implies that the original topographic variables play little role in regulating species distributions. In turn, this also is consistent with why Harms et al. [14] suggest that the original topographic variables contribute little to the species distributions. The nonlinear effect has been reported to work well for species habitat associations under the scenarios of habitat loss, patch size and isolation [34]. Because there is no such distinct abrupt change of environmental variables at this study site, the nonlinear effect does not dominantly contribute to species distribution in this study.

### Strong Neutral Spatial Effects

Our analyses based on both count data and basal area data indicate that neutral spatial effects, which are specifically represented by spatial autoregressive parameters of SAR and PCNM eigenfunctions in this study, predominantly regulate tree species distributions across multiple life stages and scales at either individual species or community levels. The spatial autoregressive parameter and PCNM eigenfunctions are both distance-limited factors, while distance is a key concept of neutral theory [35]; thus, we conclude that neutral processes are essential to the tree species distributions at the study site. In contrast to previous studies that have focused on the scale at the individual species level [4] or community level [5] or life stages at the individual species level [7] or community level [22], our study integrates all of the four scales of analysis to conclude that neutral spatial effects play a dominant role in determining species distributions. Furthermore, we verify this conclusion with both count data and basal area data.

In the present study, we extend the previously demonstrated crucial role of neutral spatial effects in shaping species distributions to multiple life stages for both basal area data and count data. Without categorizing trees into different DBH classes, many studies have verified that neutral spatial effects are the principal determinants of species distribution patterns [18], [36], [37]. He et al. [21] demonstrate that tree species distributions maintain aggregated patterns at all life stages, and we demonstrate here that neutral spatial effects are the dominant driver of tree species distributions throughout life stages. Seidler and Plotkin [38] find that seed dispersal modes are strongly correlated with the spatial aggregation of intra-species from saplings to mature trees in a 50-ha plot of Malaysian tropical forest, supporting our findings. However, this seems to vary between different forest dynamics plots. Lai et al. [7] showed that there are strong tree species habitat associations at different life stages at the individual species level. Kanagaraj et al. [22] demonstrated that habitat preference strongly determined species distributions at the juvenile stage, but neutral processes dominated the reproductive stage at the community level. As far as our study is concerned, both basal area and count data demonstrated that neutral processes overwhelmingly regulated species distributions across life stages at multiple scales at the individual species and community levels. We suggest analyzing data from multiple sites with one unified statistical method to produce more comparable results.

Legendre [12] suggests that either environmental variables or community processes may result in spatial autocorrelation, which represents the neutral spatial effect, of species distribution data. Because the two most-recognized environmental variables (topography and soil) play a limited role in determining species distributions in this study, community processes could be the crucial reasons for the spatial autocorrelation of species distribution data. Among the potential community processes, a distance-limited dispersal process has been identified as a principal process for producing tree species distributions in previous studies [18], [38]. Because both the spatial autoregressive parameters and PCNM eigenfunctions are distance-limited factors and the dispersal process is also distance-limited [39], we suggest that dispersal limitation serves as the major community process generating tree species distributions in this plot. In turn, this explains why count data and basal area data yield almost identical outcomes in the two-level analyses and is also consistent with previous studies reporting that tree species distributions are more clumped than random [11], [21], [40].

Strong neutral spatial effects are also consistent with the argument that investigating species spatial distributions without considering spatial autocorrelation may bias the analysis results [1], [13]. Kühn [41] even suggests that the analysis results may be inverted for the same data between analyses with and without the incorporation of spatial autocorrelation. Our results show that environmental variables do contribute to the tree species distributions to some extent, but both SAR and variation partitioning analyses demonstrate that neutral spatial effects are dominant in this plot.

### Count and Basal Area Data

Contrary to our expectation that the environmental variables may be more closely related to the basal area data, the pure environmental variables were identically related to basal area and count data in terms of the community composition variation partitioning results. This suggests that count data may be more appropriate for analyzing species distributions than basal area data in this plot. However, count data may not be suitable for regression analyses when species are evenly distributed across all cells in which they are present, as may occur for species with small population sizes. For example, in this study, only one individual of *Canthium simile* in DBH class 0 was counted in each cell at the 10-m spatial scale, and this resulted in an infinite value of R-squared in the SAR models.

### Effects of Spatial Scale on Species Distributions

**Figure 6:**
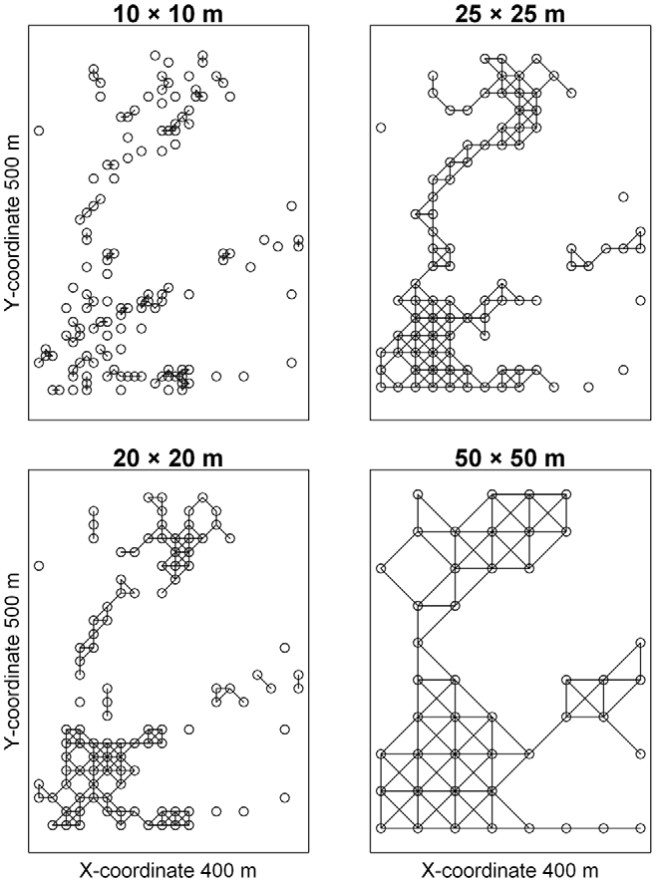
Cell connectivity at each of the four scales of cell size for *Sloanea tomentosa* in DBH class 4.

The increase in cell connectivity with cell size observed in this study may explain both the increases in the R-squared values and the total explained variation in the results of the SAR models and variation partitioning, repectively. As an example, cell connectivity clearly increased with increasing cell size for trees of *Sloanea tomentosa* in class 4 (Fig. 6). This results in decreasing p-values of *λ* with increasing cell size, except when basal area data were used at the 10-m scale. The R-squared values of the fitted models for *S. tomentosa* tend to increase with increasing cell size, except when count data are used at the 10-m scale (Fig. S13), consistent with previous work demonstrating that the variation explained by auto-Poisson regressive models when count data were used was much smaller at the 10-m scale than at the 20-m and 25-m scales in a 20-ha subtropical forest plot in southern China [4]. By contrast, in a study of the beta diversity of tree species in a 24-ha subtropical forest plot, Legendre et al. [5] found that the total explained variations in species richness and community composition varied little across sampling scales. Here, we found that the R-squared values decreased with increasing total abundance of species (Figs. 2, Figs. S3, S4, S5), in contrast to the finding of Wang et al. [4]. Because we simultaneously considered spatial scale and life stage, our analyses generated more replicates than in previous studies [4], [5], and our results may therefore more broadly reflect patterns at the individual species and community levels.

### Conclusions

In conclusion, the present study demonstrates that both lattice count data and basal area data can be reliably used to simulate the spatial distribution of tree species. Neutral spatial effects, which are specifically represented by the spatial autoregressive parameters and PCNM eigenfunctions, adequately explain the variations in both count data and basal area data at the individual species and community levels. The community processes, especially distance-limited dispersal process, may be the crucial mechanism underlying clumped patterns of species distributions. We suggest grouping trees into different DBH classes and analyzing their distributions at multiple spatial scales to enhance the applicability of the results. To achieve a broader understanding of the applicability of lattice count data and basal area data in examining species spatial distributions at both the individual species and community levels, further investigations based on large-scale plot data must be performed at additional tropical, subtropical and temperate forest sites.

## Supporting Information

Figure S1 **Patterns of median R-squared values from the fitted SAR models based on count data and basal area data at four scales of cell size, controlling for DBH class.** Circles and triangles connected by solid and dashed lines represent count data and basal area data, respectively. Bars indicate standard deviations. Classes 0 to 4 are defined as in Figure 1.
(TIF)

Figure S2 **Patterns of median R-squared values from the fitted SAR models based on count data and basal area data for five DBH classes, controlling for scale.** Circles and triangles connected by solid and dashed lines represent count data and basal area data, respectively. Classes 0 to 4 are defined as in Figure 1.
(TIF)

Figure S3 **Relationships between the R-squared values of the fitted SAR models and species total abundance for each of the 5 DBH classes at the 10-m scale.** Circles and triangles represent count data and basal area data, respectively. Classes 0 to 4 are defined as in Figure 1.
(TIF)

Figure S4 **Relationships between the R-squared values of the fitted SAR models and species total abundance for each of the 5 DBH classes at the 25-m scale.** Circles and triangles represent count data and basal area data, respectively. Classes 0 to 4 are defined as in Figure 1.
(TIF)

Figure S5 **Relationships between the R-squared values of the fitted SAR models and species total abundance for each of the 5 DBH classes at the 50-m scale.** Circles and triangles represent count data and basal area data, respectively. Classes 0 to 4 are defined as in Figure 1.
(TIF)

Figure S6 **Principal component analysis ordinations (based on matrices of transformed p-values from the SAR models) of the 14 explanatory variables and the spatial autoregressive factor λ for each of the 5 DBH classes at the 10-m scale of the count data.** Classes 0 to 4 are defined as in Figure 1. The abbreviations are defined as in Figure 3.
(TIF)

Figure S7 **Principal component analysis ordinations (based on matrices of transformed p-values from the SAR models) of the 14 explanatory variables and the spatial autoregressive factor λ for each of the 5 DBH classes at the 25-m scale of the count data.** Classes 0 to 4 are defined as in Figure 1. The abbreviations are defined as in Figure 3.
(TIF)

Figure S8 **Principal component analysis ordinations (based on matrices of transformed p-values from the SAR models) of the 14 explanatory variables and the spatial autoregressive factor λ for each of the 5 DBH classes at the 50-m scale of the count data.** Classes 0 to 4 are defined as in Figure 1. The abbreviations are defined as in Figure 3.
(TIF)

Figure S9 **Principal component analysis ordinations (based on matrices of transformed p-values from the SAR models) of the 14 explanatory variables and the spatial autoregressive factor λ for each of the 5 DBH classes at the 10-m scale of the basal area data.** Classes 0 to 4 are defined as in Figure 1. The abbreviations are defined as in Figure 3.
(TIF)

Figure S10 **Principal component analysis ordinations (based on matrices of transformed p-values from the SAR models) of the 14 explanatory variables and the spatial autoregressive factor λ for each of the 5 DBH classes at the 25-m scale of the basal area data.** Classes 0 to 4 are defined as in Figure 1. The abbreviations are defined as in Figure 3.
(TIF)

Figure S11 **Principal component analysis ordinations (based on matrices of transformed p-values from the SAR models) of the 14 explanatory variables and the spatial autoregressive factor λ for each of the 5 DBH classes at the 50-m scale of the basal area data.** Classes 0 to 4 are defined as in Figure 1. The abbreviations are defined as in Figure 3.
(TIF)

Figure S12 **Patterns of total explained variation in community composition across life stages based on count data and basal area data. The reduplicate data at each DBH class consisted of the total explained variations of the 4 scales of the variation partitioning results. Numerals 0 to 4 represent the five DBH classes which are defined as in** **Figure 1** **.**
(TIF)

Figure S13 **The p-values of λ and the R-squared values of the fitted SAR models for** ***Sloanea tomentosa*** **in DBH class 4 at each of the four spatial scales.**
(TIF)
