# Supplementary figures and images for: Strong Neutral Spatial Effects Shape Tree Species Distributions across Life Stages at Multiple Scales
Source: PLoS One. 2012 May 29;7(5):e38247. doi: 10.1371/journal.pone.0038247 (PMC3362550; doi:10.1371/journal.pone.0038247)

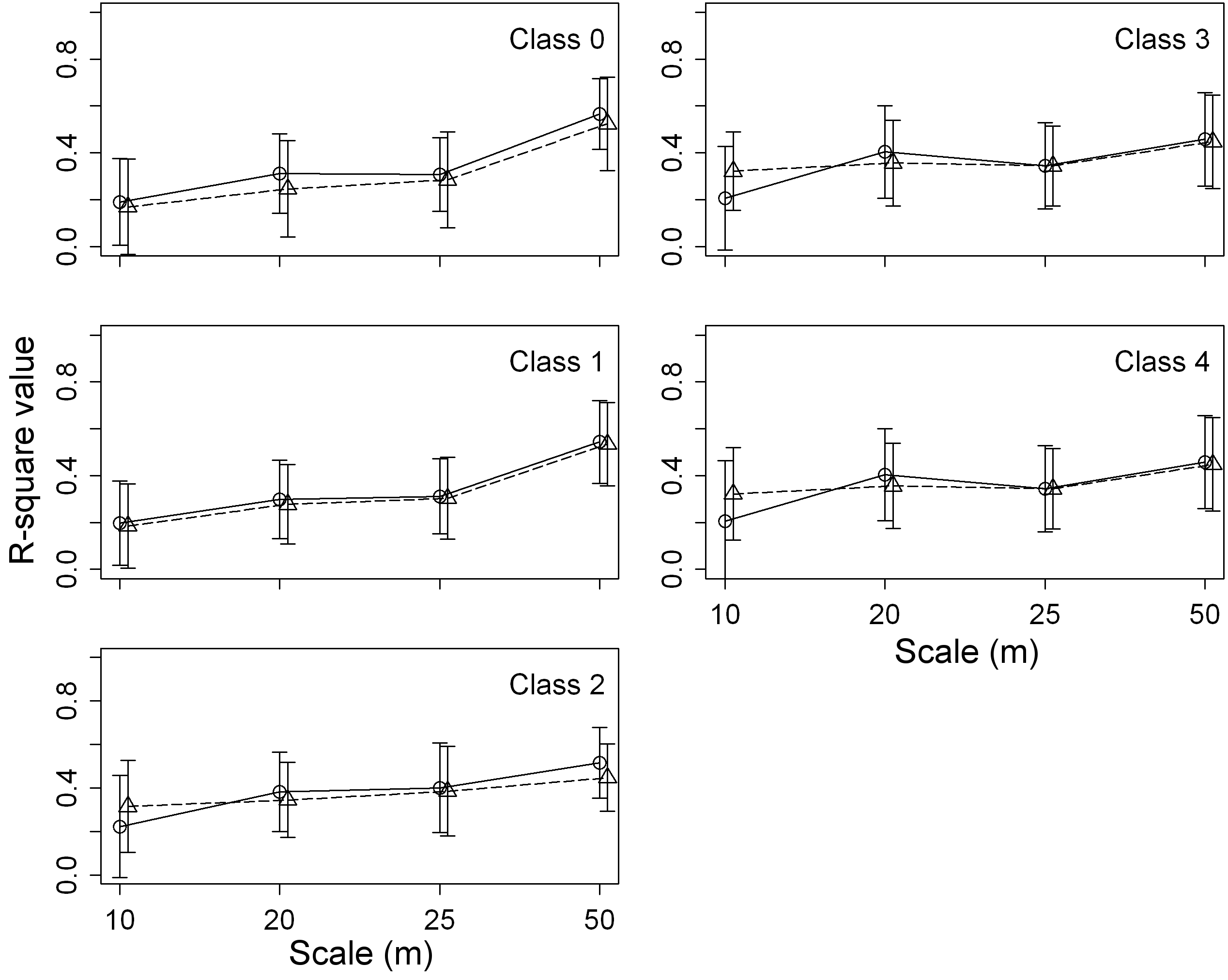

Supplement: Figure S1 — Patterns of median R-squared values from the fitted SAR models based on count data and basal area data at four scales of cell size, controlling for DBH class. Circles and triangles connected by solid and dashed lines represent count data and basal area data, respectively. Bars indicate standard deviations. Classes 0 to 4 are defined as in Figure 1. (TIF) [file pone.0038247.s001.tif]

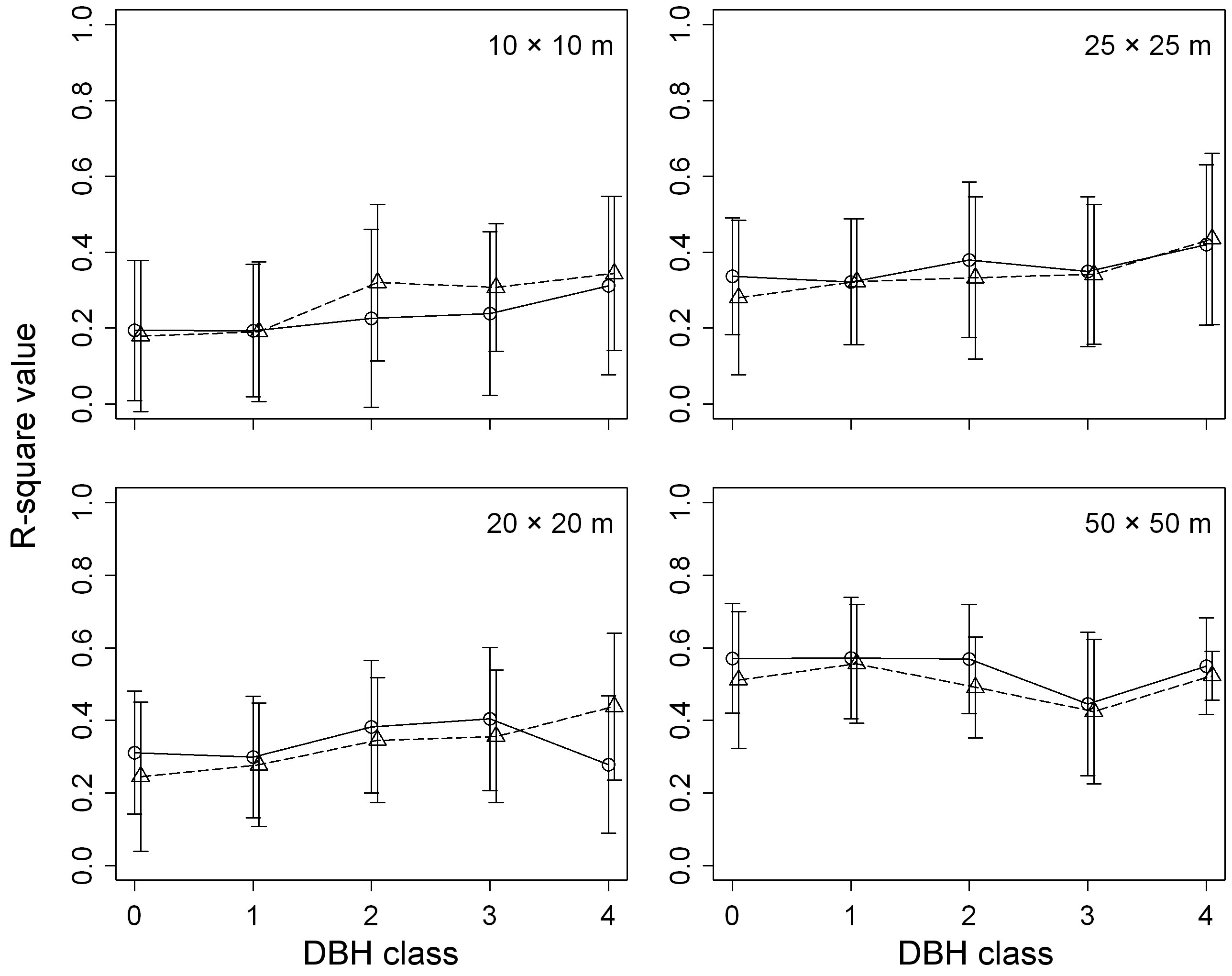

Supplement: Figure S2 — Patterns of median R-squared values from the fitted SAR models based on count data and basal area data for five DBH classes, controlling for scale. Circles and triangles connected by solid and dashed lines represent count data and basal area data, respectively. Classes 0 to 4 are defined as in Figure 1. (TIF) [file pone.0038247.s002.tif]

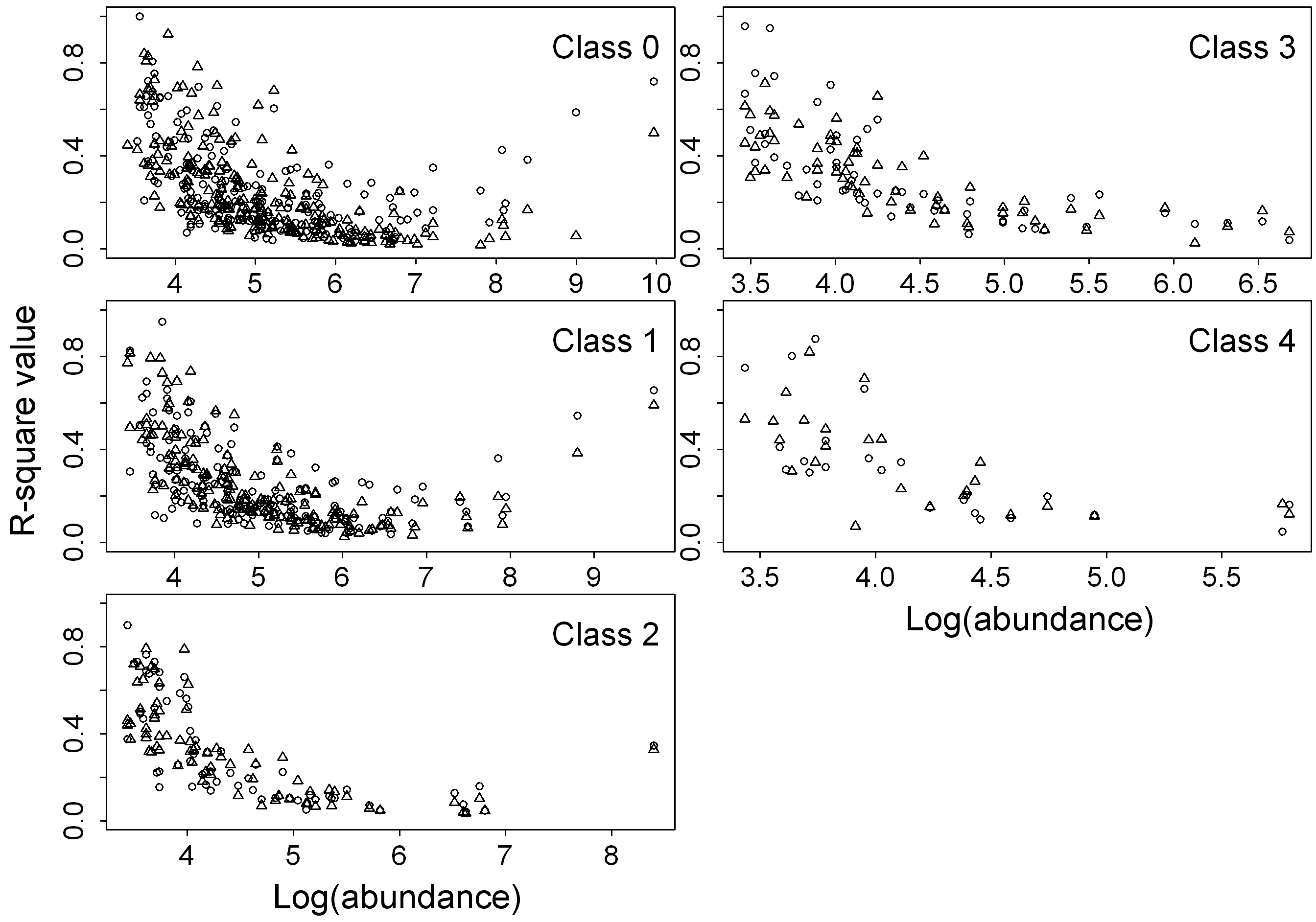

Supplement: Figure S3 — Relationships between the R-squared values of the fitted SAR models and species total abundance for each of the 5 DBH classes at the 10-m scale. Circles and triangles represent count data and basal area data, respectively. Classes 0 to 4 are defined as in Figure 1. (TIF) [file pone.0038247.s003.tif]

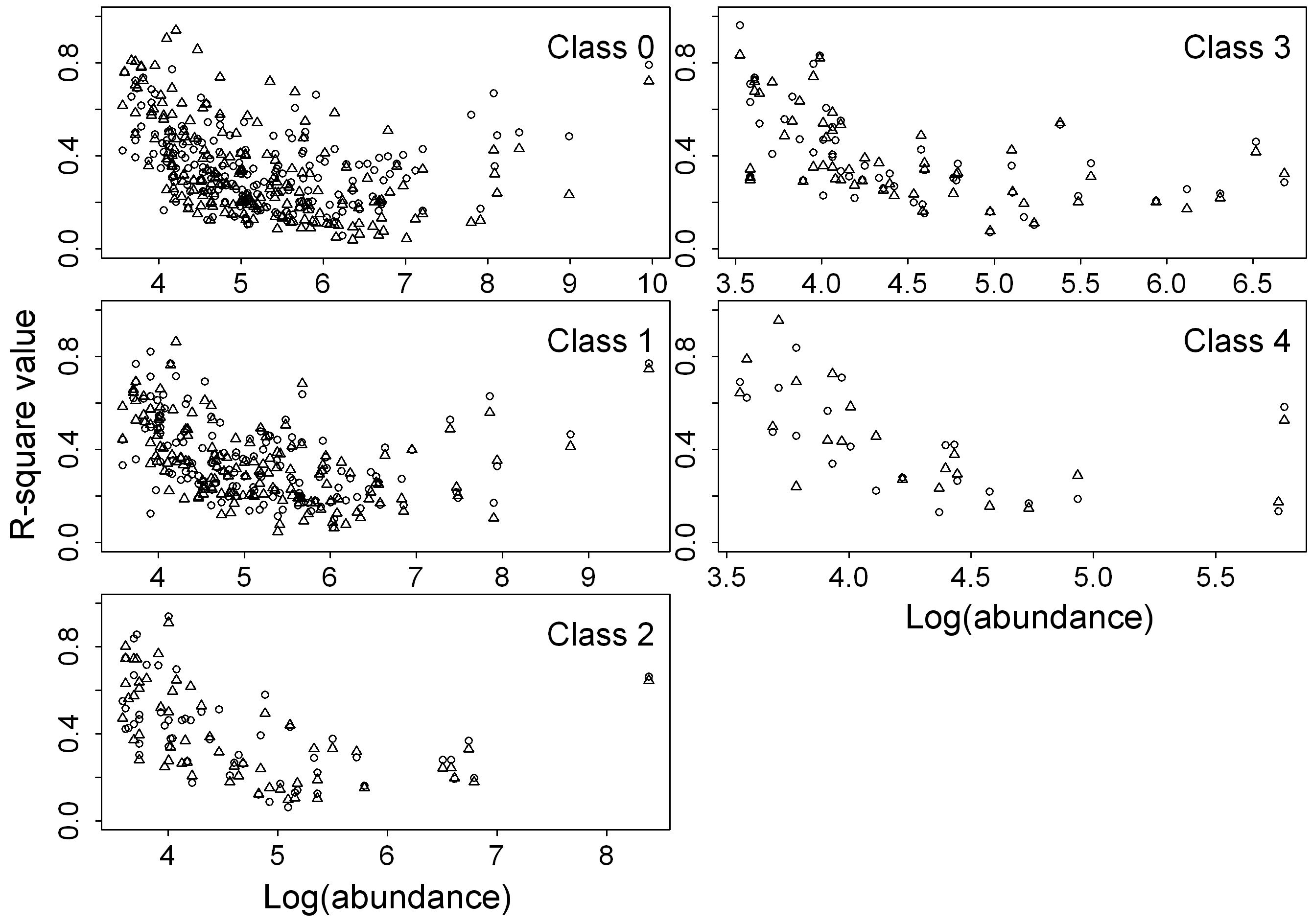

Supplement: Figure S4 — Relationships between the R-squared values of the fitted SAR models and species total abundance for each of the 5 DBH classes at the 25-m scale. Circles and triangles represent count data and basal area data, respectively. Classes 0 to 4 are defined as in Figure 1. (TIF) [file pone.0038247.s004.tif]

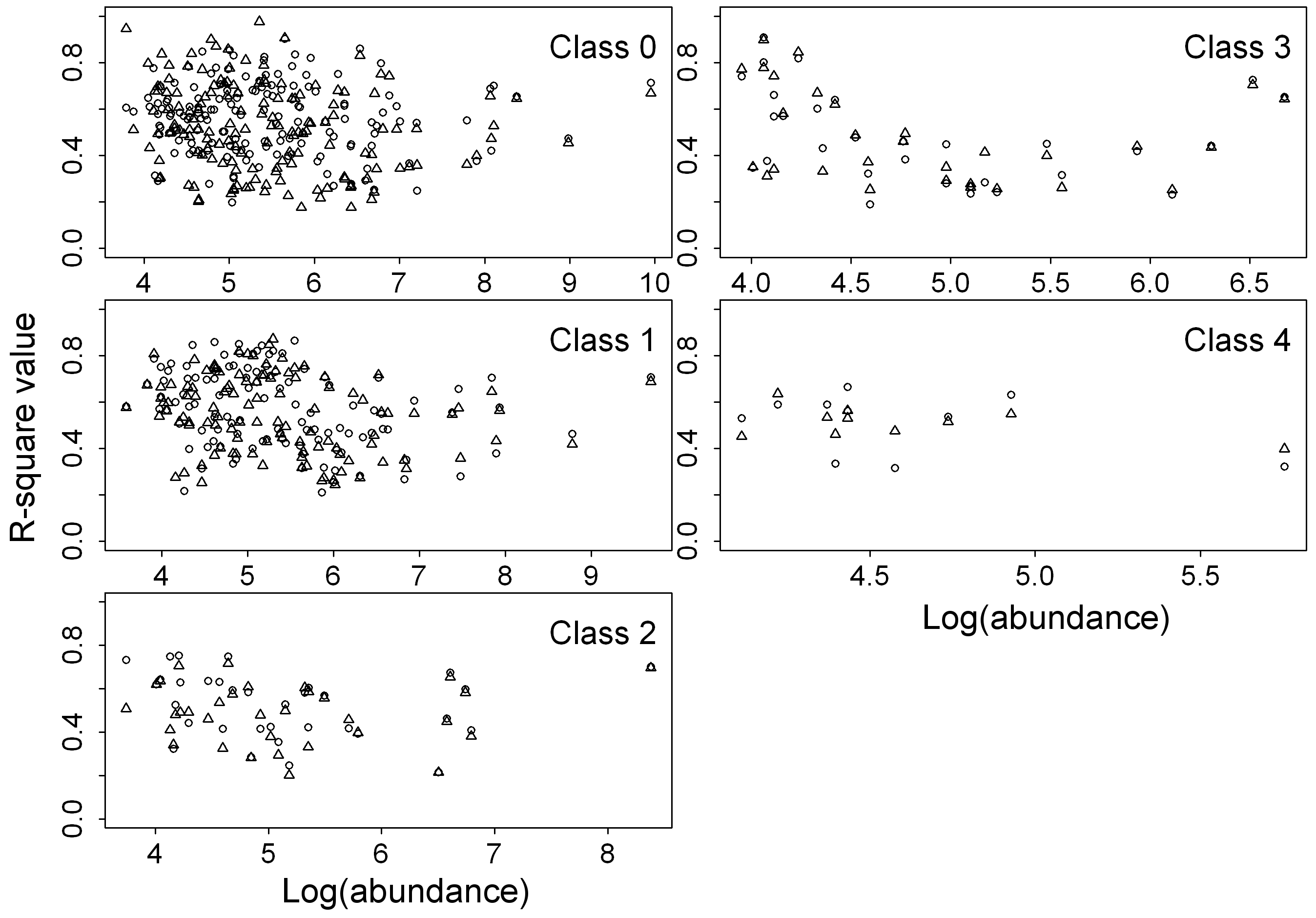

Supplement: Figure S5 — Relationships between the R-squared values of the fitted SAR models and species total abundance for each of the 5 DBH classes at the 50-m scale. Circles and triangles represent count data and basal area data, respectively. Classes 0 to 4 are defined as in Figure 1. (TIF) [file pone.0038247.s005.tif]

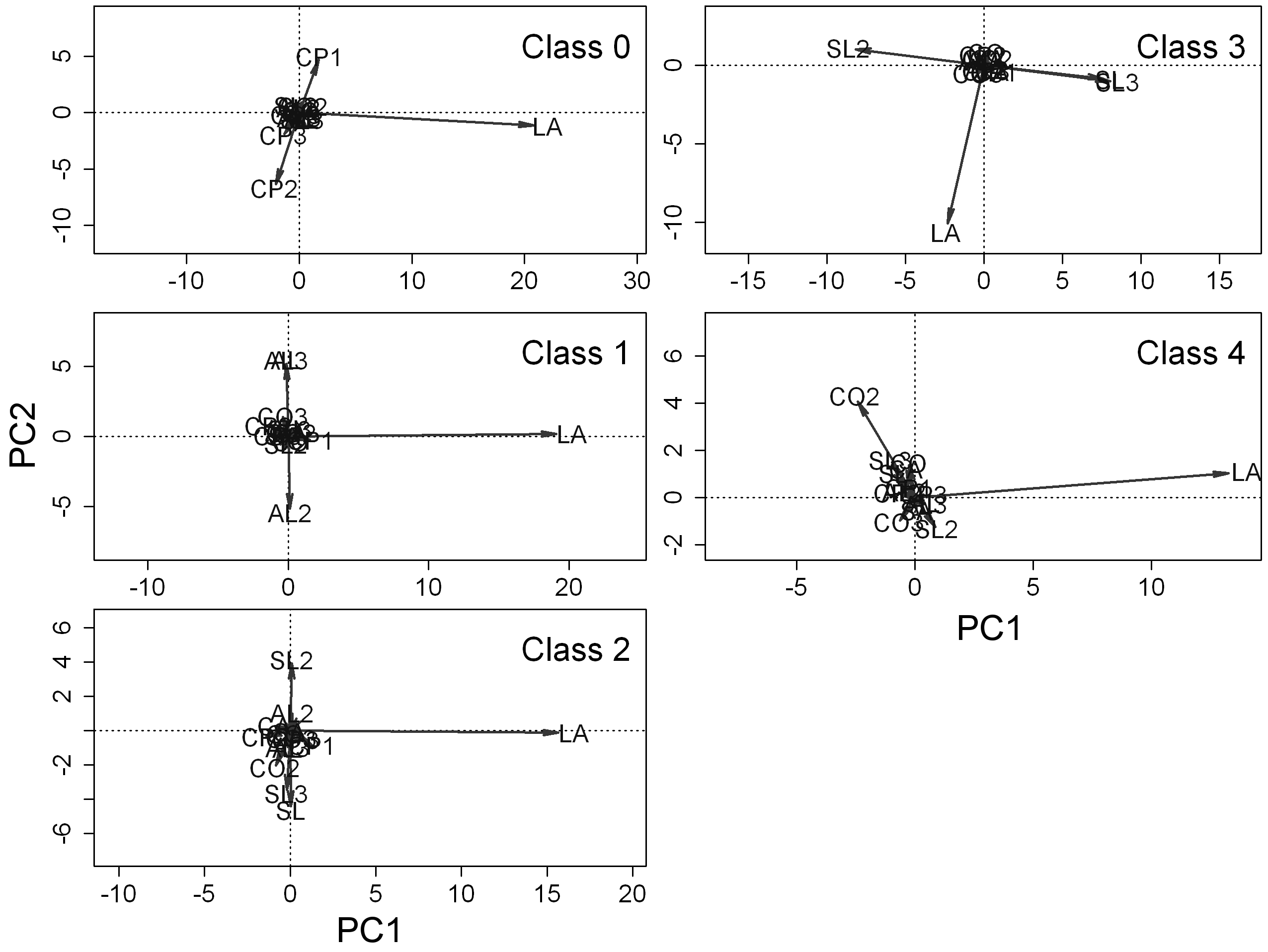

Supplement: Figure S6 — Principal component analysis ordinations (based on matrices of transformed p-values from the SAR models) of the 14 explanatory variables and the spatial autoregressive factor λ for each of the 5 DBH classes at the 10-m scale of the count data. Classes 0 to 4 are defined as in Figure 1. The abbreviations are defined as in Figure 3. (TIF) [file pone.0038247.s006.tif]

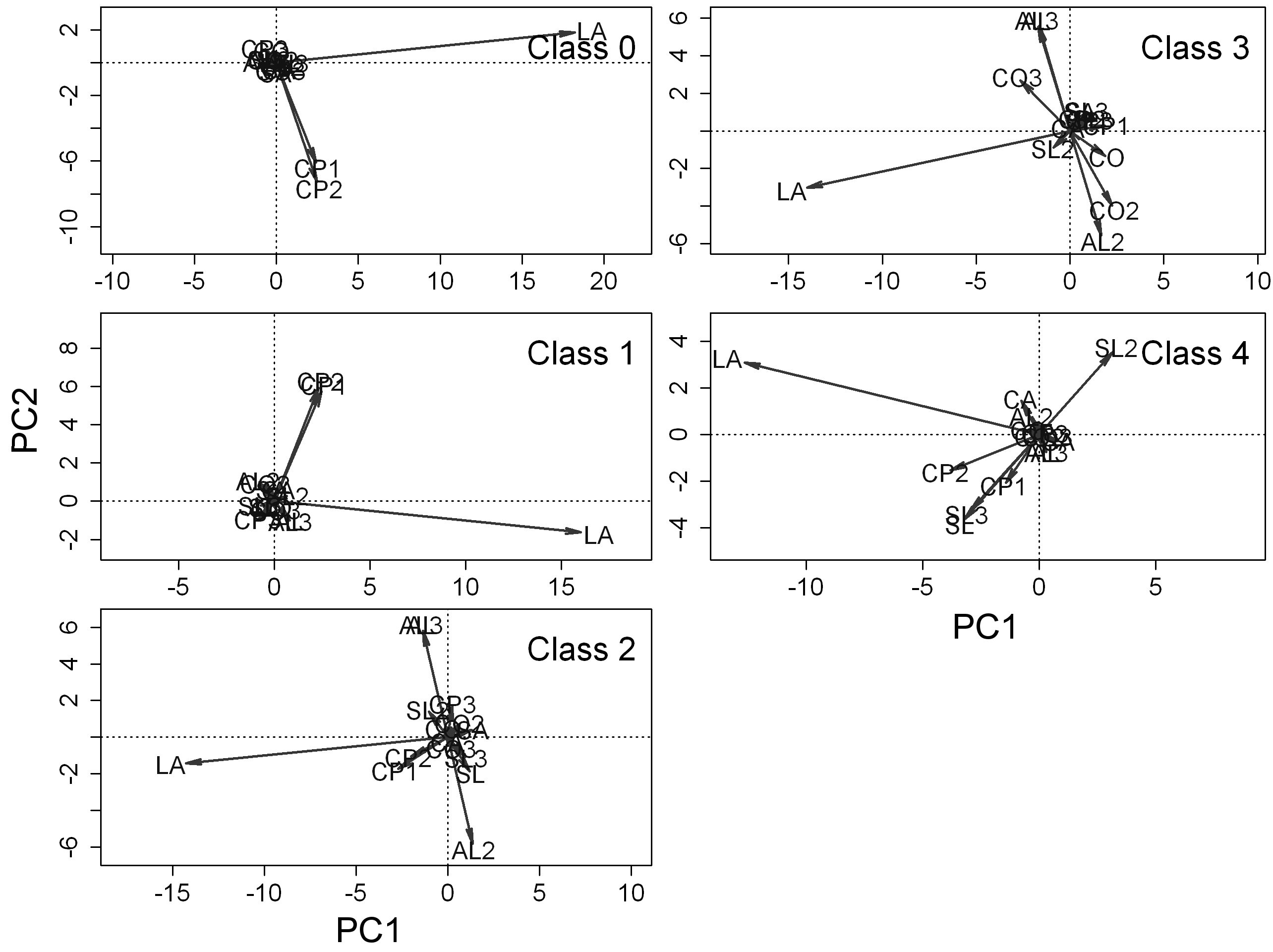

Supplement: Figure S7 — Principal component analysis ordinations (based on matrices of transformed p-values from the SAR models) of the 14 explanatory variables and the spatial autoregressive factor λ for each of the 5 DBH classes at the 25-m scale of the count data. Classes 0 to 4 are defined as in Figure 1. The abbreviations are defined as in Figure 3. (TIF) [file pone.0038247.s007.tif]

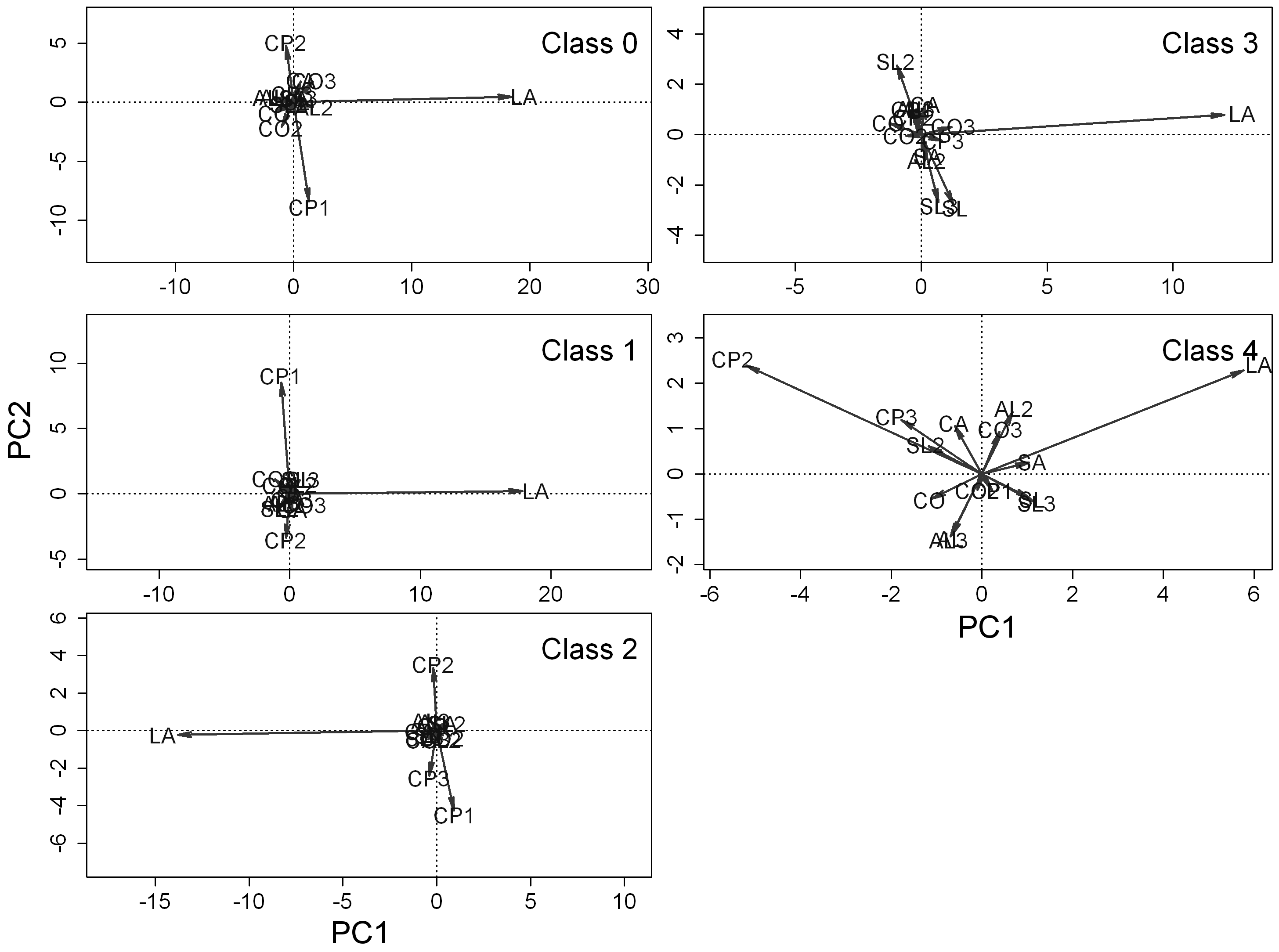

Supplement: Figure S8 — Principal component analysis ordinations (based on matrices of transformed p-values from the SAR models) of the 14 explanatory variables and the spatial autoregressive factor λ for each of the 5 DBH classes at the 50-m scale of the count data. Classes 0 to 4 are defined as in Figure 1. The abbreviations are defined as in Figure 3. (TIF) [file pone.0038247.s008.tif]

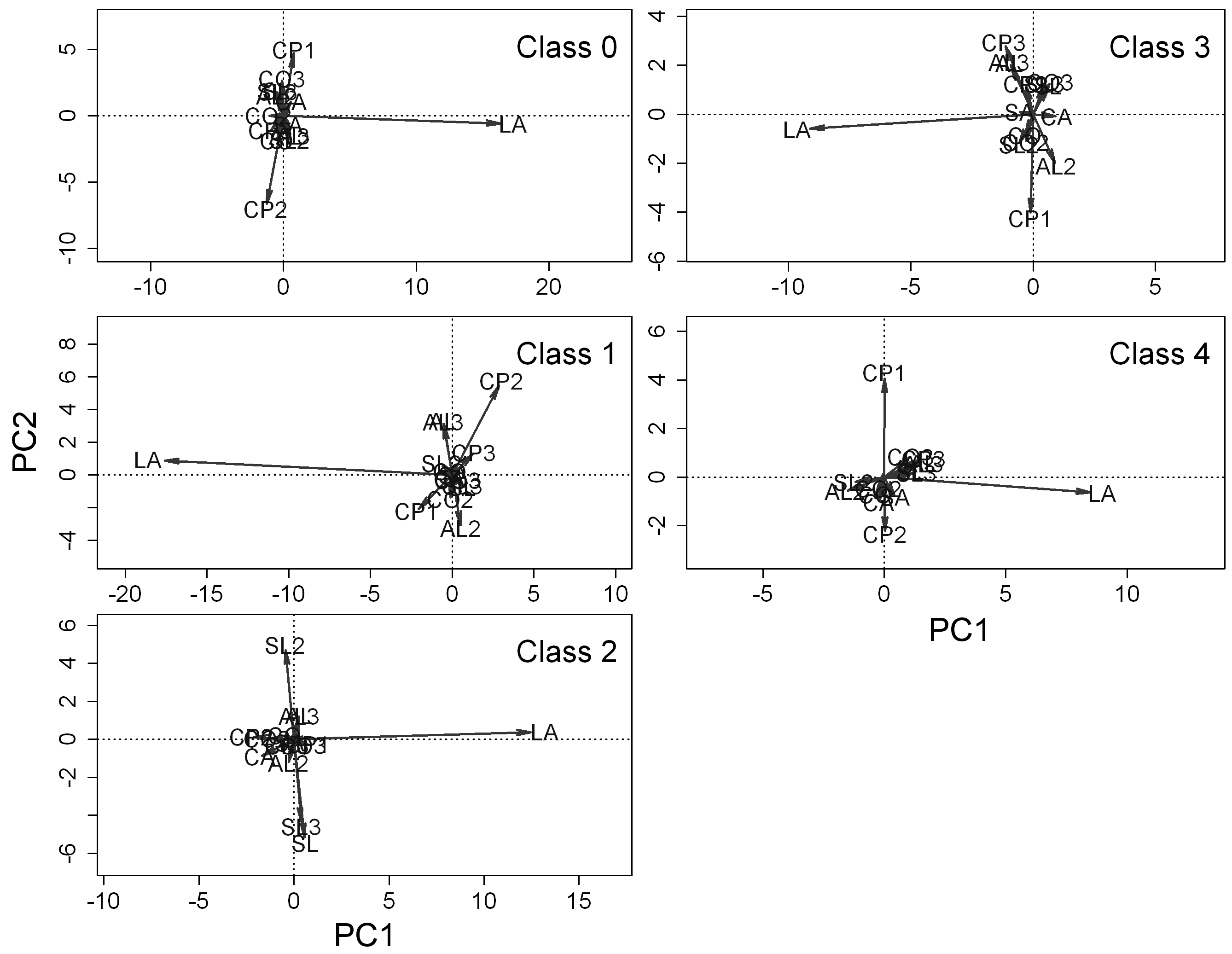

Supplement: Figure S9 — Principal component analysis ordinations (based on matrices of transformed p-values from the SAR models) of the 14 explanatory variables and the spatial autoregressive factor λ for each of the 5 DBH classes at the 10-m scale of the basal area data. Classes 0 to 4 are defined as in Figure 1. The abbreviations are defined as in Figure 3. (TIF) [file pone.0038247.s009.tif]

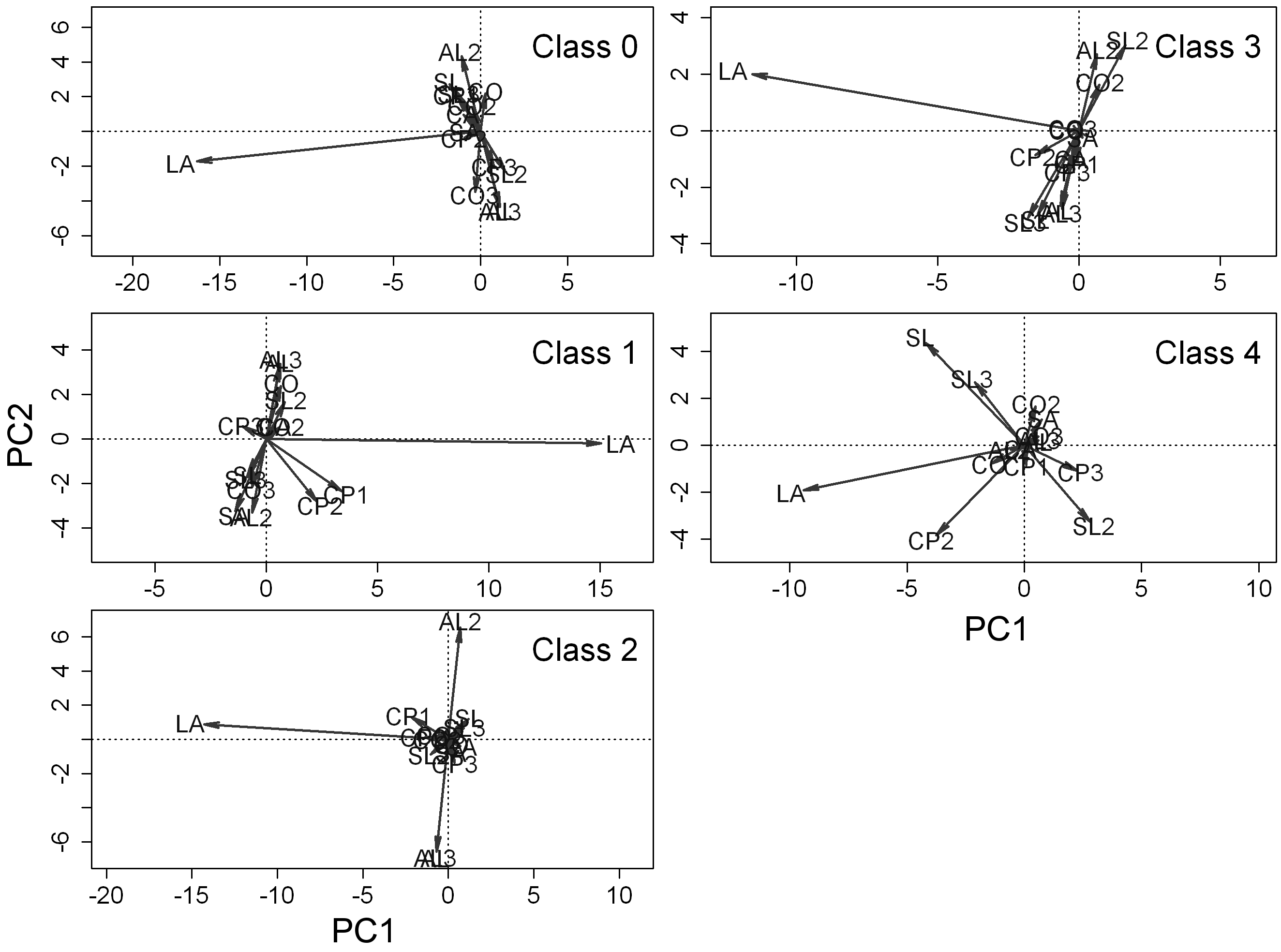

Supplement: Figure S10 — Principal component analysis ordinations (based on matrices of transformed p-values from the SAR models) of the 14 explanatory variables and the spatial autoregressive factor λ for each of the 5 DBH classes at the 25-m scale of the basal area data. Classes 0 to 4 are defined as in Figure 1. The abbreviations are defined as in Figure 3. (TIF) [file pone.0038247.s010.tif]

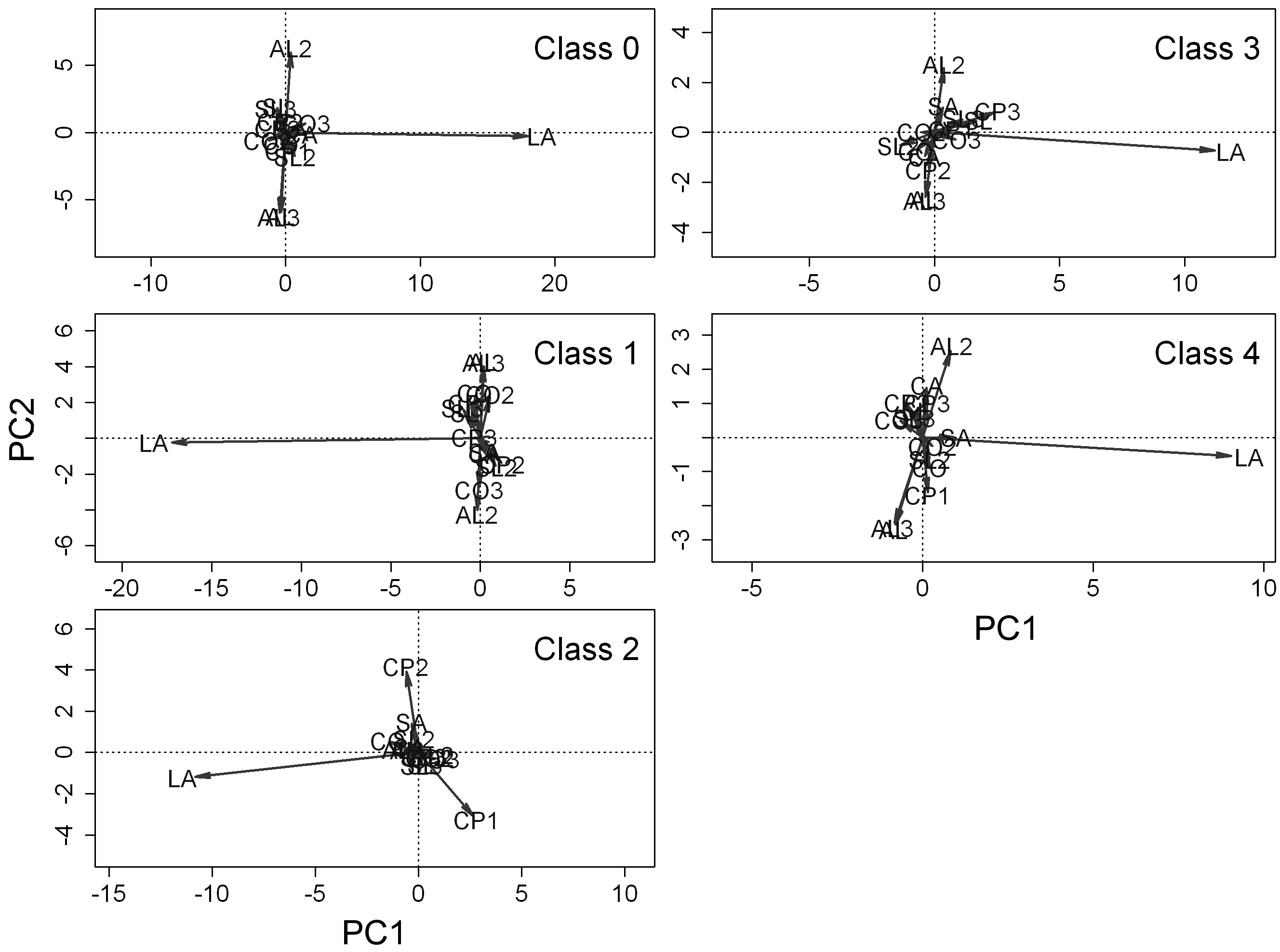

Supplement: Figure S11 — Principal component analysis ordinations (based on matrices of transformed p-values from the SAR models) of the 14 explanatory variables and the spatial autoregressive factor λ for each of the 5 DBH classes at the 50-m scale of the basal area data. Classes 0 to 4 are defined as in Figure 1. The abbreviations are defined as in Figure 3. (TIF) [file pone.0038247.s011.tif]

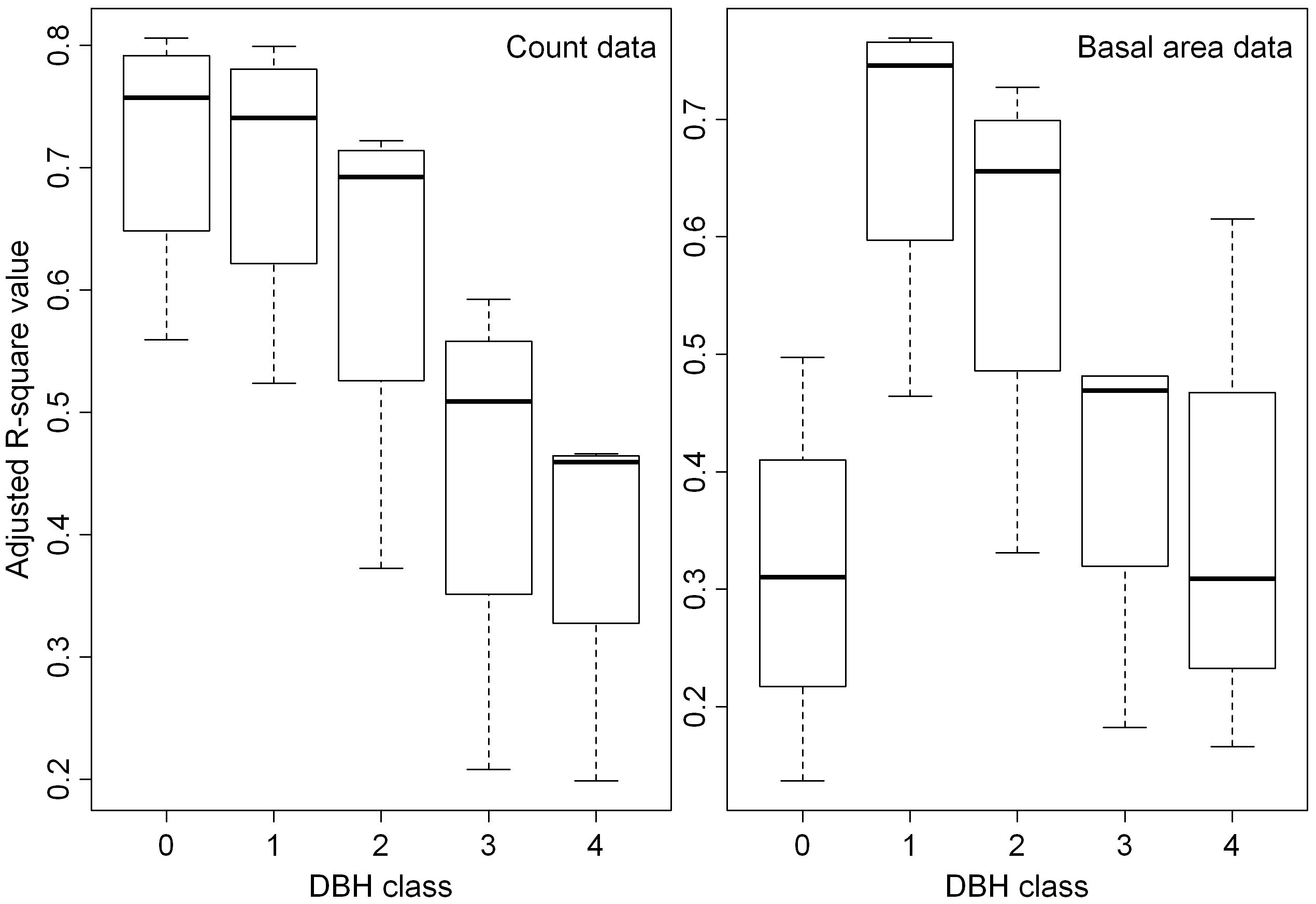

Supplement: Figure S12 — Patterns of total explained variation in community composition across life stages based on count data and basal area data. The reduplicate data at each DBH class consisted of the total explained variations of the 4 scales of the variation partitioning results. Numerals 0 to 4 represent the five DBH classes which are defined as in Figure 1 . (TIF) [file pone.0038247.s012.tif]

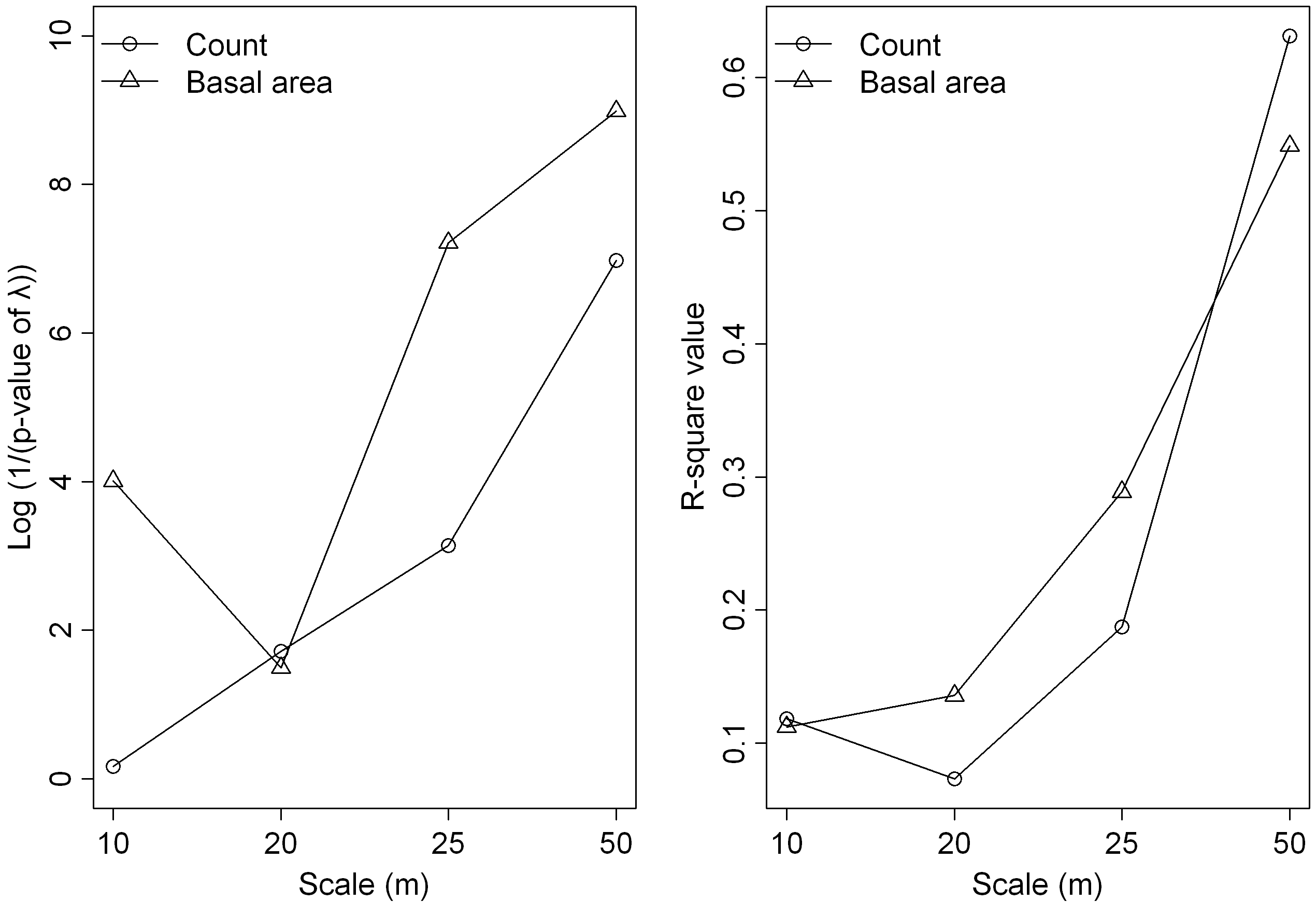

Supplement: Figure S13 — The p-values of λ and the R-squared values of the fitted SAR models for Sloanea tomentosa in DBH class 4 at each of the four spatial scales. (TIF) [file pone.0038247.s013.tif]
